# Differential Regulation of *PIWI-LIKE 2* Expression in Primordial Germ Cell Tumor Cell Lines by Promoter Methylation

**DOI:** 10.3389/fgene.2018.00375

**Published:** 2018-09-20

**Authors:** Maria Giebler, Thomas Greither, Hermann M. Behre

**Affiliations:** Center for Reproductive Medicine and Andrology, Martin Luther University of Halle-Wittenberg, Halle, Germany

**Keywords:** epigenetics, *PIWI-LIKE 2*, germ cell tumors, promoter methylation, spermatogenesis

## Abstract

*PIWI-LIKE 2*, a member of the ARGONAUTE protein family, is exclusively expressed in pre-pachytene and pachytene stages of spermatogenesis. *PIWI-LIKE 2* acts in the germ cell development and the silencing of retrotransponsons to maintain the genomic integrity and stem cell character. In the present study we investigated DNA methylation as potential mechanism for the regulation of human *PIWI-LIKE 2* expression in cell lines related to spermatozoa precursor cells. We detected a high methylation of the *PIWI-LIKE 2* promoter in TCam-2 cells, while in NT2/D1 cells the promoter was hypomethylated. Concordantly, *PIWI-LIKE 2* expression is higher in NT2/D1 cells than in TCam-2 cells. By demethylation of the promoter with 5′-Aza-2′-deoxycytidine, *PIWI-LIKE 2* expression in TCam-2 was increased, while in NT2/D1 no alterations in *PIWI-LIKE 2* expression could be detected. In conclusion, we analyzed the DNA methylation driving *PIWI-LIKE 2* expression in undifferentiated germ cell tumors and demonstrated an epigenetic basis for *PIWI-LIKE 2* expression in this cell type.

## Introduction

Spermatogenesis is a highly coordinated process that involves mitotic and meiotic divisions, as well as cellular differentiation to produce mature spermatozoa from undifferentiated germline stem cells. Sperm development is associated with the establishment of extensive chromatin and epigenetic changes. This process allows genomic chemical modifications that affect gene expression without altering the underlying nucleotide sequence (Cui et al., 2016). Epigenetic modifications are characterized by the regulation of non-coding RNA, chromatin remodeling, histone modifications and DNA methylation (Stuppia et al., 2015). DNA methylation occurs at the 5′-position of cytosine residues, typically in the context of CpG dinucleotides, which are associated with promoter regions of genes in about 60–80% (Seisenberger et al., 2013; Liyanage et al., 2014). Methylation of CpG sites leads to transcriptional gene silencing in consequence of an altered condensation status of the chromatin. Genomic methylation profiling analysis reveals cell type specific methylation patterns, which result in cell-type specific differential gene expressions and thus differentially regulated tissue-specific processes. Methylation marks for proper male gametogenesis are established during genomic reprogramming in early embryonic development and indicate an exclusive genetic profile of male germ cells compared to somatic tissues (Santos et al., 2002; Bourc’his and Bestor, 2004).

Another factor for successful male germ cell development is the coordinated and timely expression of the members of the *PIWI-LIKE* gene family, the germ line specific subclade of the Argonaute proteins. These proteins are characterized by the presence of their evolutionarily conserved PAZ (PIWI-ARGONAUTE-ZWILLE) and PIWI (P-element induced wimpy testis) domains (Cox et al., 1998; Cerutti et al., 2000). These structural features function in transcriptional and post-transcriptional control by binding to small RNAs. Thereby PAZ domain is responsible for 3’-end recognition of the bound small RNA, whereas the PIWI domain is involved in mRNA target binding and cleavage (Song et al., 2004; Jinek and Doudna, 2009). PIWI-LIKE proteins are known to bind a distinct class of small RNAs. These small RNAs, called piRNAs (piwi-interacting RNAs), are frequently 24–31 nt in length, map to distinct genomic regions and share a high preference for 5′ Uridine (Aravin et al., 2006, 2007; Grivna et al., 2006). In germline development, PIWI/piRNA complexes mediate the self-renewal of germline stem cells and maintain genomic integrity through suppression of mobile genetic elements and retrotransposons, such as long interspersed nuclear elements-1 (*LINE-1*) (Unhavaithaya et al., 2009; Marchetto et al., 2013).

The human PIWI subfamily comprises *HIWI (PIWI-LIKE 1)*, *HILI (PIWI-LIKE 2), HIWI3 (PIWI-LIKE 3)* and *HIWI2 (PIWI-LIKE 4). PIWI-LIKE 2* is exclusively expressed in spermatogonia and pre-meiotic spermatocytes (Sasaki et al., 2003). However, it has been demonstrated to be temporarily activated in somatic cells in response to DNA damages (Lim et al., 2013) Furthermore, *PIWI-LIKE 2* reveals ectopic expression in several tumor entities, and its intragenically activated products, such as PL2L60A, are expressed in various types of tumor cell lines (Ye et al., 2010; Gainetdinov et al., 2014). Potential regulation mechanisms of *PIWI-LIKE 2* expression are scarcely investigated. Normally, *MILI* (the murine homolog of *HILI/PIWI-LIKE 2*) is exclusively expressed in the spermatogonia and spermatocytes (Sasaki et al., 2003) and in the female oocytes and supporting cells (Lim et al., 2013).

The aim of this study was to identify epigenetic mechanisms that may underlie the differential expression of *PIWI-LIKE 2* in germ line and somatic tissues by analyzing the basal promoter methylation of *PIWI-LIKE 2* and the effects of a modification of this methylation by 5′-Aza-2′-deoxycytidine treatment in two different *in vitro* models, TCam-2 and NT2/D1 cells.

## Materials and Methods

### Cell Culture

Cell lines used for the experiments were the following: TCam-2, a human seminoma cell line with characteristics similar to spermatogonia; and NT2/D1, a human teratocarcinoma cell line with characteristics and gene expression profiles similar to cultured human embryonic stem cells. TCam-2 cells were grown in RPMI 1640 GlutaMAX^TM^ supplemented with 10% fetal calf serum (FCS) and 1% penicillin/streptomycin. NT2/D1 cells were cultivated in DMEM GlutaMAX^TM^ supplemented with 10% FCS and 1% penicillin/streptomycin. The cells were grown as monolayer at 37°C in a 5% CO2 humidified incubator.

### 5′-Aza-2′-Deoxycytidine Treatment

For 5′-Aza-2′-deoxycytidine treatment TCam-2 and NT2/D1 cells were plated in a concentration of 4×10^5^ cell in corresponding cultivation medium supplemented with 5′ μM or 10 μM 5′-Aza-2′-deoxycytine (Sigma Aldrich, Taufkirchen, Germany) for 72 h, afterward DNA, RNA and protein was isolated as described. DMSO served as vehicle control.

### DNA Isolation

Genomic DNA was isolated from each cell line with and without treatment of 5′-Aza-2′-deoxycytidine. DNA was isolated using MasterPure^TM^ Complete DNA Purification Kit (Epicentre, United States) according to the manufacturer’s instructions. Extracted DNA was quantified using the BioPhotometer (Eppendorf, Hamburg, Germany) and the purity was determined by OD260/OD280 ratio.

### Bisulfite Sequencing PCR (BSP)

Five hundred nanograms genomic DNA was treated with sodium bisulfite using the EpiTect Bisulfite Kit (Qiagen, Hilden, Germany). Bisulfite treatment converts unmethylated cytosines into uracils while leaving methylated cytosines unmodified. During PCR amplification the generated uracils are converted to thymidine. The methylation specific primers were designed using MethPrimer software^1^. DNA Primers are listed in **Table 1**. 50 ng sodium bisulfite treated DNA was used for each PCR reaction. PCR was performed under following conditions: 95°C for 5 min, followed by 35 cycles of 95°C for 30 s, 60°C for 30 s and 72°C for 30 s. Purified PCR products were cloned into a pCR2.1 vector and ten clones from each sample were submitted to SEQLAB Sequencing Laboratories (Göttingen, Germany) for sequencing analysis. DNA methylation data were analyzed using BiQ Analyzer Software (Max Planck Institute, Munich, Germany).

**Table 1:** Primer list.

Name	Sequence (5′-3′)	Application
**Bisulfite sequencing PCR (BSP)**
PIWI2 Me_Ins1 fw	GGTAGGAATGGGGTAAGTTAATT	Promoter methylation studies
PIWI2 Me_Ins1 rv	CACATACTCCAAAACCAATTTC	
PIWI2 Me_Ins2 fw	GATGGGTTAATTAGATAGTTTGTT	
PIWI2 Me_Ins2 rv	CTAAACACCTTCTTAAAACC	
**Cloning and Sequencing**
pCR2.1 -TOPO fw	CAGGAAACAGCTATGAC	Cloning and sequencing
pCR2.1 -TOPO rv	GTAAAACGACGGCCAG	
pGL4.10[luc2] fw	CTAGCAAAATAGGCTGTCCC	
pGL4.10[luc2] rv	GCCCTTCTTAATGTTTTTG	
**Luciferase assay**
PIWI2 Prom_ A fw	AAACTCGAGTGGTGCCCAGGGTATTTGGAGTC	Promoter activation studies
PIWI2 Prom_ A rv	TTTAAGCTTTGGCATGCTCCAGGGCCAATTTC	
PIWI2 Prom_ B fw	AAACTCGAGTGGTGCCCAGGGTATTTGGAGTC	
PIWI2 Prom_ B rv	TTTAAGCTTTGGTAGCGATACAGGTGGTGAAA	
PIWI2 Prom_ C fw	AAAGGTACCTGGTGTGGGAGAGGGATGCAGTTA	
PIWI2 Prom_ C rv	TTTAAGCTTTGGTAGCGATACAGGTGGTGAAA	
PIWI2 Prom_ D fw	AAACTCGAGTGGTGTGGGAGAGGGATGCAGTTA	
PIWI2 Prom_ D rv	TTTAAGCTTTGGAACCGGGGCCAGTACTCA	
PIWI2 Prom_ E fw	AAACTCGAGTGGTGTATCGCAATCCTCTTAA	
PIWI2 Prom_ E rv	TTTAAGCTTTGGGCCAGGGGTTCTATCTCCTC	
PIWI2 Prom_ F fw	AAAGGTACCTGGACAGGTCTTGTGGCCAATGG	
PIWI2 Prom_ F rv	TTTAAGCTTTGGGTAGCAGATACTTGGCTGTC	

### Cloning and Sequencing

Different *PIWI-LIKE 2* upstream regulating regions were generated using PCR (Primers listed in **Table 1**). Products were ligated into a pCR2.1 vector according to the manufacturer’s protocol (Life Technologies, Germany). Plasmids were checked for the correct inserts using BigDye^®^ Terminator v1.1 Cycle Sequencing Kit by ZMG. 5 μg Plasmid DNA, including Basic pGL4.10[luc2] vector, were digested with Hind III and XhoI (1 U/μl) for 2h at 37°C. Digest was analyzed using 1% agarose gel and purified using the DNA Gel Extraction Kit (Thermo Fisher, Germany). Fragments were subcloned into pGL4.10[luc2] vector according to the manufacturer’s instructions and validated using sequencing analysis.

### *In vitro* Methylation

One microgram Xho I/HindIII digested DNA was treated for 4 h with M.SssI methylase (4 U/μl, NEB) according to the manufacturer’s protocol. *In vitro* methylation was controlled by BSTU I digest at 60°C for 1 h. Fragments were ligated into a pGL4.10[luc2] vector (Promega, Mannheim, Germany) and used for luciferase reporter assays.

### Quantitative RT-PCR

Total RNA was extracted from cell lines using TRIzol according to the manufacturer’s instructions. 1 μg of the total RNA was reverse transcribed using RevertAid H Minus First Strand cDNA Synthesis Kit (Life Technologies, Germany). Quantitative PCR was performed using the MyiQ Real Time PCR Detection System (Biorad, Germany). *PIWI-LIKE 2* (Hs01032719_m1) TaqMan Primers targeting exon 4–5 were used for the detection of *PIWI-LIKE 2* expression. *GAPDH* was used as reference gene (fw: 5′-CAAGGTCATCCATGACAACTTTG-3′ and rv: 5′-GTCCACCACCCTGTTGCTGTAG-3′). The data were normalized to *GAPDH* levels, and levels of *PIWI-LIKE 2* mRNA were determined using the 2^-ΔCt^ method.

### Protein Isolation

Proteins were isolated from TCam-2 cells and NT2/D1 cells by RIPA buffer 50 mM TRIS–HCl pH 8, 150 mM NaCl, 1% NP-40, 0.5% sodium deoxycholate, 0.1% SDS + 1 unit of protease inhibitor cocktail (Roche, Mannheim, Germany). The lysates were incubated for 10 min at 4°C, followed by a 10 min centrifugation step at 13,000 rpm, 4°C. The supernatant was used for western blot analysis. The protein concentration was determined by BCA Protein Assay Kit (Biorad, Germany).

### Western Blot

For protein analysis, we used the Novex Mini Cell Electrophoresis (Life Technologies, Germany) and Mini *Trans*-Blot system (BioRad, Germany). Protein was prepared by standard protocols and electrophoresed at 125 mA for 60 min. Gels were blotted onto a polyvinylidene fluoride membrane in a BioRad blotting chamber for 2 h at 200 V at 4°C according to published protocols. After the membrane had been blocked in PBSTM (phosphate-buffered saline, 0.1% v/v Tween 20, 5% low fat milk powder), it was incubated in a solution containing primary antibodies raised against PIWI-LIKE 2 (ab181340, 1:5000, Abcam, Germany) or ß-ACTIN (AC-15 mouse antibody, 1:10,000, Sigma-Aldrich, Germany) at 4°C overnight. Secondary antibody (anti-rabbit-horseradish peroxidase [HRP], DAKO, Germany) incubation in a 1:10,000 dilution was applied for 1 h at RT. Finally, the membrane was incubated in 1 ml Amersham ECL prime Western Blot detection system and the signal was detected by using Kodak X-Ray film (Kodak, Germany).

### Luciferase Reporter Assay

The *PIWI-LIKE 2* fragments used in the luciferase reporter assays were amplified by PCR (primers including XhoI and HindIII recognition sites listed in **Table 1**). PCR was carried out at 95°C for 2 min, followed by 35 cycles of 95°C for 30 s, 60°C for 30 s and 72°C for 2 min. pGL4.10[luc2] vector (Promega, Mannheim, Germany) containing the luciferase gene under control of the *PIWI-LIKE 2* fragments was transfected in 1 × 10^4^ TCam2 or NT2/D1 cells at a ratio of 3:2 (μl transfection reagent: μg DNA). FuGeneHD (Roche, Germany) was used as transfection reagent. Additionally pGL4.10[luc2] *PIWI-LIKE 2* constructs methylated by M.SssI were transfected. Transfection of empty pGL4.10[luc2] vector and untreated cells were used as additional controls. Transfections were conducted as co-transfections with pGL4.74 empty vector containing a constitutive expressed Renilla fly as normalization control in a ratio of 1:50 (Renilla fly: Luciferase fly). 48 h later, cells were lysed for 20 min in 50 μl 1× lysis buffer. Luciferase activity was measured after addition of the luciferase assay buffer by a luminometer in a 96-well plate.

## Results

### Identification of *PIWI-LIKE 2* Promoter

**FIGURE 1:**
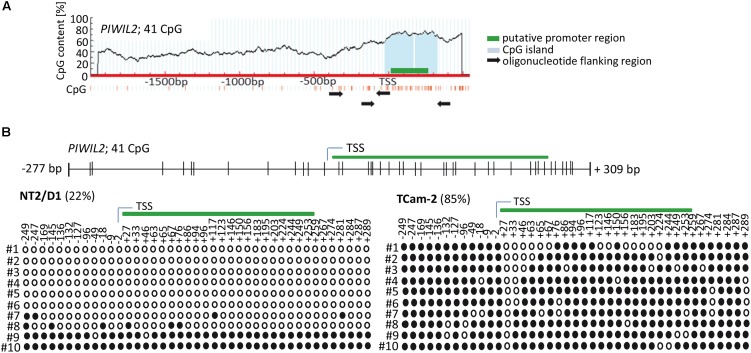
*PIWI-LIKE 2* promoter analysis **(A)** schematic diagram of CpG dinucleotide density across the genomic *PIWI-LIKE 2* locus. Green box indicates putative promoter region. Light blue box shows the predicted CpG island. Black arrows designate the location of the oligonucleotides used for bisulfite sequencing PCR. **(B)** Upper panel shows the location of the investigated CpG dinucleotides according to the transcription start site (TSS) of *PIWI-LIKE 2*. Lower panel shows basic *PIWI-LIKE 2* methylation level of the investigated CpG dinucleotides in NT2/D1 and TCam-2 cells. Filled circles indicate methylated CpGs, empty circles indicate demethylated CpGs. #1-10 refers to the different clones analyzed per *PIWI-LIKE 2* promoter fragment.

Firstly, we analyzed the region upstream of *PIWI-LIKE 2* transcription start site (TSS) to identify a putative promoter as well as target sites for CpG methylations that might contribute to gene expression regulation. Using PromoterScan1.7 Software^2^ we identified the region +4 bp to +254 bp adjacent to the *PIWI-LIKE 2* TSS containing a putative promoter. Further analysis of the selected region using MethPrimer Software^3^ showed the occurrence of 41 CpG dinucleotides set in a CpG island. All of these CpG sites were located within the -300 to +300 bp region relative to the TSS of *PIWI-LIKE 2* (**Figure 1A**). We investigated the methylation status of the *PIWI-LIKE 2* promoter using bisulfite sequencing. The basal levels of *PIWI-LIKE 2* promoter methylation differ in the analyzed cell lines. In the seminoma cell line TCam-2, the *PIWI-LIKE 2* promoter is heavily methylated (85%), whereas in the pluripotent embryonal carcinoma cell line NT2/D1, the *PIWI-LIKE 2* promoter exhibits a low promoter methylation (22%) (**Figure 1B**).

### *PIWI-LIKE 2* Expression Is Induced by 5AzadC in TCam-2

**FIGURE 2:**
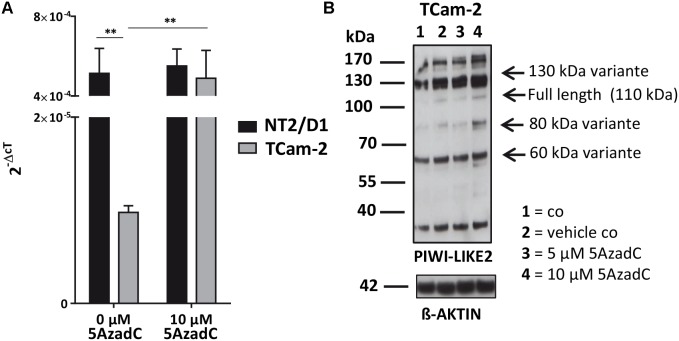
*PIWI-LIKE 2* mRNA and protein expression analysis **(A)**. qRT-PCR was performed without and with 5AzadC treatment after 72 h in NT2/D1 and TCam-2 cells. The data shown are means ± SD of three independent experiments. ^∗^*P* < 0.05, Student’s *t*-test. **(B)** Western Blot analysis of PIWI-LIKE 2 was conducted after treatment of TCam-2 cells without and with 5AzadC for 72 h. Untreated cells and DMSO treated cells served as control. ß-ACTIN was the loading control. ^∗∗^*P* < 0.01.

**FIGURE 3:**
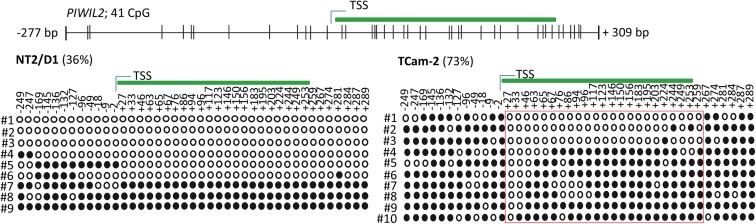
*PIWI-LIKE 2* promoter CpG methylation analysis. Upper panel shows the location of the investigated CpG dinucleotides according to the TSS of *PIWI-LIKE 2*. Lower panel shows *PIWI-LIKE 2* methylation level of the investigated CpG dinucleotides in NT2/D1 and TCam-2 cells after treatment with 5AzadC for 72h. Red boxes indicate CpG dinucleotides with prominent changes in methylation status. Filled circles indicate methylated CpG’s, empty circles indicate demethylated CpGs. #1-10 refers to the different clones analyzed per *PIWI-LIKE 2* promoter fragment.

Next, we tested whether the basal *PIWI-LIKE 2* mRNA expression correlates to the CpG promoter methylation in both analyzed human cell lines. mRNA expression was analyzed using qRT-PCR, and cT (cycle threshold) values from three independent experiments were taken to assess mean *PIWI-LIKE 2* mRNA expressions calculated by the 2^-ΔCt^ method with normalization to *GAPDH* mRNA expression. We found that the expression of *PIWI-LIKE 2* mRNA was 52 times higher in NT2/D1 cells (2^-ΔCt^mean = 5.14^∗^10^-4^) compared to TCam-2 cells (2^-ΔCt^mean = 9.77^∗^10^-6^; *p* = 0.01) (**Figure 2A**). This difference in mRNA expression could be explained by a different methylation status of both cell lines. Furthermore, the induction of *PIWI-LIKE 2* expression by the demethylating agent 5′-Aza-2′-deoxycytidine (5AzadC) was analyzed in the used cell lines. Treatment of TCam-2 cells with 10 μM 5AzadC led to a 50 times enhanced expression of *PIWI-LIKE 2* mRNA (2^-ΔCt^mean = 4.89^∗^10^-4^; *p* = 0.027) compared to the basic mRNA expression. Protein expression of *PIWI-LIKE 2* was measured in TCam2 after 72 h incubation with and without 5AzadC (**Figure 2B**). *PIWI-LIKE 2* protein expression was increased with rising 5AzadC concentration (5 μM–10 μM; lane 3,4) compared to the vehicle control (lane 2). An increase of protein products with 130 kDa, 110 kDa, 80 kDa, and 60 kDa could be detected. Different molecular weights could point out on *PIWI-LIKE 2* splice variants of different length and modifications. Neither *PIWI-LIKE 2* mRNA expression (2^-ΔCt^mean = 5.51E-04) nor protein expression (not shown) was changed after treatment with 5AzadC in NT2/D1 cell line. Next, we examined the impact of 5AzadC on *PIWI-LIKE 2* promoter methylation status in NT2/D1 and TCam-2 cell lines. Treatment with 5AzadC led to a decrease in methylation of the CpG dinucleotides located within the -130 to +65 bp region in TCam-2 and reduced the overall methylation of the investigated promoter segment from 85 to 73% (**Figure 3**). NT2/D1 cell line exhibited no decrease in methylation status of the analyzed promoter site. These results suggest that basal methylation of *PIWI-LIKE 2* depends on the origin of the cell line. Treatment of cells with 5-AzadC allows a partial demethylation of the *PIWI-LIKE 2* promoter. Of interest, knockdown of *PIWI-LIKE 2* in either NT2/D1 or TCam-2 cells resulted in a significant reduction of proliferation (-48.2 and –19.6%, respectively, compared to untreated control) and cell vitality (-62.9 and -30.6%, respectively) and in a significant induction of apoptosis (+1351% and + 716%, respectively; see **Supplementary Figure 1**, **2**).

### *In vitro* Activation of *PIWI-LIKE 2* Promoter

**FIGURE 4:**
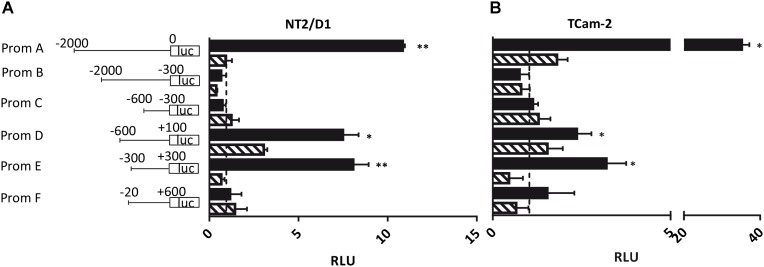
Luciferase assay in NT2/D1 **(A)** and TCam-2 cells **(B)**. The luciferase reporter assays were performed 48 h after transfection with the indicated *PIWI-LIKE 2* promoter fragments of different length and location and a renilla transfection control plasmid, which was co-transfected. An empty luciferase vector served as control and was set to an expression of 1 (dashed line). Respective *PIWI-LIKE 2* promoter constructs after *in vitro* methylation were also transfected. The data shown are means ± SD of three independent experiments. ^∗^*P* < 0.05, ^∗∗^*P* < 0.01, Student’s *t*-test.

Next, we addressed the question whether the *PIWI-LIKE 2* promoter can be activated *in vitro* and if the activation could be silenced by methylation of CpG-sites within this sequence. Therefore, six promoter fragments of different length and location were generated (A-F; see **Figure 4**). We assumed the region 2000 bp downstream of the TSS of the *PIWI-LIKE 2* full length variant to potentially regulate *PIWI-LIKE 2* transcription (Fragment A). Furthermore, we designed shortened promoter fragments containing up to 600 bp downstream of the TSS. Promoter fragment E represented the region around the predicted CpG island and putative promoter site (-300 bp to +300 bp). Fragments were cloned 5′ of a luciferase gene and transfected into the embryonal carcinoma cell line NT2/D1 and the seminoma cell line TCam-2. A constitutive Renilla luciferase expressing pGL4.74 plasmid was co-transfected for normalization. Luciferase activity was strongly induced by the *PIWI-LIKE 2* full length promoter fragment compared to the empty reporter construct. After 48 h of transfection there was a 35fold (*p* = 0.007) increase in TCam-2 and 10fold activation in NT2/D1 (*p* = 0.001). The shortened promoter fragment D leaded to an induction as well. There was a three fold induction in TCam-2 (*p* = 0.049) and a 6.5fold increasing of luciferase activity in NT2/D1 (*p* = 0.034). The CpG island containing fragment E showed an eightfold activation of luciferase expression in NT2/D1 (*p* = 0.0002) and a threefold activation in TCam-2 (*p* = 0.014). Transfection of *in vitro* methylated promoter fragments resulted in a reduced luciferase activity for all fragments.

## Discussion

DNA methylation is highly dynamic in mammalian germ cells during development. Maternal and paternal genomes are differentially marked and must be properly reprogrammed before the primordial germ cell enters the germinal ridge (Santos et al., 2002; Sasaki and Matsui, 2008; Messerschmidt et al., 2014). DNA methylation as epigenetic modification of the mammalian genome has widespread influences on gene expression. *PIWI-LIKE* genes are known to be involved in germ cell specification and maturation. *MIWI2* (the murine homolog of human *PIWI-LIKE 4)* is expressed in the pre-pachytene phase of spermatogenesis during the period of *de novo* methylation. It has been detected in the nucleus, where it acts directly on transposable elements repression on DNA level via the piRNA metabolic process. Inactivation of *MIWI2* leads to male sterility due to an early meiotic arrest, which correlates with retrotransposon derepression (Carmell et al., 2007; Aravin et al., 2008; Fazio et al., 2011). *PIWI-LIKE 1* and *PIWI-LIKE 2* associate with primary piRNAs in the cytoplasm and are required for the *PIWI-LIKE 4* nuclear localization and association with secondary piRNAs antisense (Aravin et al., 2006, 2007; Reuter et al., 2011). The piRNA process acts upstream of known mediators of the DNA methylation. Besides their function in transposable elements repression, piRNAs are probably involved in other processes during meiosis, such as translation regulation (Thomson and Lin, 2009).

*PIWI-LIKE 2* is completely repressed in somatic cells and, during development, silenced at post-meiotic stages. In this study, we wanted to investigate promoter DNA methylation as potential mechanism for expression control of *PIWI-LIKE 2*. A high density of CpG sites spanning the region at about -300 bp to +300 bp relative to the *PIWI-LIKE 2* full length TSS makes an epigenetic regulation conceivable. *PIWI-LIKE 2* promoter is hypomethylated (22%) in NT2/D1, but highly methylated in TCam-2. Concomitantly, NT2/D1 shows a higher basal mRNA expression. These results suggest CpG methylation status of *PIWI-LIKE 2* correlates with its expression. Neither CpG methylation nor mRNA and protein expression was changed in NT2/D1 after demethylation treatment via 5′-Aza-2′-deoxycytidine (5AzadC). This indicates an open chromatin status on the *PIWI-LIKE 2* promoter in this cell line, which is not altered and enables a higher *PIWI-LIKE 2* expression in comparison to TCam-2. However, it is of note that although most of the clones analyzed in NT2/D1 were hypomethylated, 2 of 10 (basal methylation state) or 3 of 9 (after 5AzadC treatment) were hypermethylated in a way which is comparable to T-Cam2. This observation seems to stand in contradiction to the proposed hypomethylation of the *PIWI-LIKE 2* promoter in cell types or lines with a higher differentiation potential. However, one may speculate that during the cultivation and treatment of the NT2/D1, in single cells the differentiation process was induced unintentionally. The observed hypermethylation of the PIWI-LIKE 2 promoter may in this context be another indication for silencing of *PIWI-LIKE 2* expression as an very early event during germ cell differentiation.

In TCam-2, 5AzadC treatment leads to a significant increase of *PIWI-LIKE 2* mRNA and protein, but a comparable low overall promoter demethylation (85–73%). Interestingly, almost only CpG dinucleotides spanning the *PIWI-LIKE 2* promoter region from -130 nt to +65 nt are partially demethylated. Either this region is relevant for *PIWI-LIKE 2* expression regulation or a splice variant could potentially be activated, which is not regulated by the investigated 41 CpG islands and has another promoter site. Gainetdinov et al. (2014) demonstrated the presence of 60 kDa (PL2L60A) and 80 kDa (PL2L80A) isoforms of PIWI-LIKE 2 in testicular cancer cell lines including NT2/D1. Furthermore, they identified alternative TSSs within the *PIWI-LIKE 2* sequence. These were mapped to exon 5 and exon 7 and can be activated *in vitro*. Beyond, there is evidence for the existence of *PIWI-LIKE 2* splice variants by isoforms with 50 kDa (PL2L50) and 40 kDa (PL2L40) (Ye et al., 2010). Recently, an intragenic promoter in intron 10 of the *PIWI-LIKE 2* genomic sequence regulating a 60 kDa isoform was identified in human cells and verified in luciferase reporter assays (Liu et al., 2017). Here, we show that the full length *PIWI-LIKE 2* promoter and promoter fragments surrounding the TSS of full length *PIWI-LIKE 2* are able to drive luciferase expression in human cell lines. The activation was markedly reduced after *in vitro* methylation of these fragments. The data indicate that in humans DNA methylation is able to induce epigenetically silencing of *PIWI-LIKE 2* expression. Furthermore, it suggests the region around the TSS and exon 1 is subject of epigenetic regulation.

Our data exhibit a high mRNA expression but low overall CpG demethylation in TCam-2. Furthermore, we observed a decreased proliferation and cell vitality and an increased apoptosis induction upon suppression of *PIWI-LIKE 2* expression in both NT2/D1 and TCam-2. Analogously, insufficient *PIWI-LIKE 2* expression is associated with male infertility in mouse and human (Kuramochi-Miyagawa et al., 2004; Kuramochi-Miyagawa et al., 2008; Heyn et al., 2012). In human, the dysfunction was associated with the hypermethylation of *PIWI-LIKE 2* promoter and its interacting factor *TDRD1* and resulted in a disrupted production of piRNAs and a hypomethylation of the *LINE-1* repetitive sequences in patients affected with spermatogenic arrest (Heyn et al., 2012). An proposed association of single nucleotide polymorphisms (SNPs) in the *PIWI-LIKE 2* gene with spermatogenic failure (Gu et al., 2010) has not been re-analyzed in other male infertility patient cohorts so far.

## Conclusion

In conclusion, *PIWI-LIKE 2* is essential for the germline integrity and self-renewal of stem cells. Therefore, it may be an interesting target for the prediction of fertilization rates and embryo development in assisted reproductive techniques (ART). Thus, the methylation profile of *PIWI-LIKE 2* and its corresponding expression could potentially provide further predictive information for clinical decisions.

## Author Contributions

MG performed the experiments, analyzed the data, and drafted the manuscript. TG and HB conceived the study, assisted in drafting the manuscript, and reviewed the data and the manuscript.

## Conflict of Interest Statement

The authors declare that the research was conducted in the absence of any commercial or financial relationships that could be construed as a potential conflict of interest.

## Footnotes

1 http://www.urogene.org/methprimer/

2 www-bimas.cit.nih.gov/molbio/proscan/

3 http://www.urogene.org/methprimer/

## References

[B1] Aravin A. A. Gaidatzis D. Pfeffer S. Lagos-Quintana M. Landgraf P. Iovino N. (2006). A novel class of small RNAs bind to MILI protein in mouse testes. *Nature* 442 203–207. 10.1038/nature04916 16751777

[B2] Aravin A. A. Sachidanandam R. Bourc’his D. Schaefer C. Pezic D. Fejes-Toth K. (2008). A piRNA pathway primed by individual transposons is linked to de novo DNA methylation in mice. *Mol. Cell* 31 785–799. 10.1016/j.molcel.2008.09.003 18922463 PMC2730041

[B3] Aravin A. A. Sachidanandam R. Girard A. Fejes-Toth K. Hannon G. J. (2007). Developmentally regulated piRNA clusters implicate MILI in transposon control. *Science* 316 744–747. 10.1126/science.1142612 17446352

[B4] Bourc’his D. Bestor T. H. (2004). Meiotic catastrophe and retrotransposon reactivation in male germ cells lacking Dnmt3L. *Nature* 431 96–99. 10.1038/nature02886 15318244

[B5] Carmell M. A. Girard A. van de Kant H. J. G. Bourc’his D. Bestor T. H. de Rooij D. G. (2007). MIWI2 is essential for spermatogenesis and repression of transposons in the mouse male germline. *Dev. Cell* 12 503–514. 10.1016/j.devcel.2007.03.001 17395546

[B6] Cerutti L. Mian N. Bateman A. (2000). Domains in gene silencing and cell differentiation proteins: the novel PAZ domain and redefinition of the Piwi domain. *Trends Biochem. Sci.* 25 481–482. 10.1016/S0968-0004(00)01641-8 11050429

[B7] Cox D. N. Chao A. Baker J. Chang L. Qiao D. Lin H. (1998). A novel class of evolutionarily conserved genes defined by piwi are essential for stem cell self-renewal. *Genes Dev.* 12 3715–3727. 10.1101/gad.12.23.3715 9851978 PMC317255

[B8] Cui X. Jing X. Wu X. Yan M. Li Q. Shen Y. (2016). DNA methylation in spermatogenesis and male infertility. *Exp. Ther. Med.* 12 1973–1979. 10.3892/etm.2016.3569 27698683 PMC5038464

[B9] Fazio S. Bartonicek N. Di Giacomo M. Abreu-Goodger C. Sankar A. Funaya C. (2011). The endonuclease activity of Mili fuels piRNA amplification that silences LINE1 elements. *Nature* 480 259–263. 10.1038/nature10547 22020280

[B10] Gainetdinov I. V. Skvortsova Y. V. Stukacheva E. A. Bychenko O. S. Kondratieva S. A. Zinovieva M. V. (2014). Expression profiles of PIWI-LIKE 2 short isoforms differ in testicular germ cell tumors of various differentiation subtypes. *PLoS One* 9:e112528. 10.1371/journal.pone.0112528 25384072 PMC4226551

[B11] Grivna S. T. Beyret E. Wang Z. Lin H. (2006). A novel class of small RNAs in mouse spermatogenic cells. *Genes Dev.* 20 1709–1714. 10.1101/gad.1434406 16766680 PMC1522066

[B12] Gu A. Ji G. Shi X. Long Y. Xia Y. Song L. (2010). Genetic variants in Piwi-interacting RNA pathway genes confer susceptibility to spermatogenic failure in a Chinese population. *Hum. Reprod.* 25 2955–2961. 10.1093/humrep/deq274 20940137

[B13] Heyn H. Ferreira H. J. Bassas L. Bonache S. Sayols S. Sandoval J. (2012). Epigenetic disruption of the PIWI pathway in human spermatogenic disorders. *PLoS One* 7:e47892. 10.1371/journal.pone.0047892 23112866 PMC3480440

[B14] Jinek M. Doudna J. A. (2009). A three-dimensional view of the molecular machinery of RNA interference. *Nature* 457 405–412. 10.1038/nature07755 19158786

[B15] Kuramochi-Miyagawa S. Kimura T. Ijiri T. W. Isobe T. Asada N. Fujita Y. (2004). Mili, a mammalian member of piwi family gene, is essential for spermatogenesis. *Development* 131 839–849. 10.1242/dev.00973 14736746

[B16] Kuramochi-Miyagawa S. Watanabe T. Gotoh K. Totoki Y. Toyoda A. Ikawa M. (2008). DNA methylation of retrotransposon genes is regulated by Piwi family members MILI and MIWI2 in murine fetal testes. *Genes Dev.* 22 908–917. 10.1101/gad.1640708 18381894 PMC2279202

[B17] Lim S. L. Tsend-Ayush E. Kortschak R. D. Jacob R. Ricciardelli C. Oehler M. K. (2013). Conservation and expression of PIWI-interacting RNA pathway genes in male and female adult gonad of amniotes. *Biol. Reprod.* 89:136. 10.1095/biolreprod.113.111211 24108303

[B18] Liu S.-S. Liu N. Liu M.-Y. Sun L. Xia W.-Y. Lu H.-M. (2017). An unusual intragenic promoter of PIWI-LIKE 2 contributes to aberrant activation of oncogenic PL2L60. *Oncotarget* 8 46104–46120. 10.18632/oncotarget.17553 28545024 PMC5542253

[B19] Liyanage V. R. Jarmasz J. S. Murugeshan N. Del Bigio M. R. Rastegar M. Davie J. R. (2014). DNA modifications: function and applications in normal and disease States. *Biology* 3 670–723. 10.3390/biology3040670 25340699 PMC4280507

[B20] Marchetto M. C. N. Narvaiza I. Denli A. M. Benner C. Lazzarini T. A. Nathanson J. L. (2013). Differential L1 regulation in pluripotent stem cells of humans and apes. *Nature* 503 525–529. 10.1038/nature12686 24153179 PMC4064720

[B21] Messerschmidt D. M. Knowles B. B. Solter D. (2014). DNA methylation dynamics during epigenetic reprogramming in the germline and preimplantation embryos. *Genes Dev.* 28 812–828. 10.1101/gad.234294.113 24736841 PMC4003274

[B22] Reuter M. Berninger P. Chuma S. Shah H. Hosokawa M. Funaya C. (2011). Miwi catalysis is required for piRNA amplification-independent LINE1 transposon silencing. *Nature* 480 264–267. 10.1038/nature10672 22121019

[B23] Santos F. Hendrich B. Reik W. Dean W. (2002). Dynamic reprogramming of DNA methylation in the early mouse embryo. *Dev. Biol.* 241 172–182. 10.1006/dbio.2001.0501 11784103

[B24] Sasaki H. Matsui Y. (2008). Epigenetic events in mammalian germ-cell development: reprogramming and beyond. *Nat. Rev. Genet.* 9 129–140. 10.1038/nrg2295 18197165

[B25] Sasaki T. Shiohama A. Minoshima S. Shimizu N. (2003). Identification of eight members of the Argonaute family in the human genome. *Genomics* 82 323–330. 10.1016/S0888-7543(03)00129-0 12906857

[B26] Seisenberger S. Peat J. R. Hore T. A. Santos F. Dean W. Reik W. (2013). Reprogramming DNA methylation in the mammalian life cycle: building and breaking epigenetic barriers. *Philos. Trans. R. Soc. Lond. B Biol. Sci.* 368:20110330. 10.1098/rstb.2011.0330 23166394 PMC3539359

[B27] Song J.-J. Smith S. K. Hannon G. J. Joshua-Tor L. (2004). Crystal structure of Argonaute and its implications for RISC slicer activity. *Science* 305 1434–1437. 10.1126/science.1102514 15284453

[B28] Stuppia L. Franzago M. Ballerini P. Gatta V. Antonucci I. (2015). Epigenetics and male reproduction: the consequences of paternal lifestyle on fertility, embryo development, and children lifetime health. *Clin. Epigenetics* 7:120. 10.1186/s13148-015-0155-4 26566402 PMC4642754

[B29] Thomson T. Lin H. (2009). The biogenesis and function of PIWI proteins and piRNAs: progress and prospect. *Annu. Rev. Cell Dev. Biol.* 25 355–376. 10.1146/annurev.cellbio.24.110707.175327 19575643 PMC2780330

[B30] Unhavaithaya Y. Hao Y. Beyret E. Yin H. Kuramochi-Miyagawa S. Nakano T. (2009). MILI, a PIWI-interacting RNA-binding protein, is required for germ line stem cell self-renewal and appears to positively regulate translation. *J. Biol. Chem.* 284 6507–6519. 10.1074/jbc.M809104200 19114715 PMC2649106

[B31] Ye Y. Yin D.-T. Chen L. Zhou Q. Shen R. He G. (2010). Identification of PIWI-LIKE 2 -like (PL2L) proteins that promote tumorigenesis. *PLoS One* 5:e13406. 10.1371/journal.pone.0013406 20975993 PMC2958115

