# Supplementary material for: Differential Regulation of PIWI-LIKE 2 Expression in Primordial Germ Cell Tumor Cell Lines by Promoter Methylation
Source: Front Genet. 2018 Sep 20;9:375. doi: 10.3389/fgene.2018.00375 (PMC6158399; doi:10.3389/fgene.2018.00375)
Supplement: Supplementary file 1 [file Table_1.docx]

Supplementary Material

**Differential regulation of *PIWI-LIKE 2* expression in primordial germ cell tumor cell lines by promoter methylation**

**Maria Giebler, Thomas Greither, Hermann M. Behre***

*** Correspondence:** Corresponding Author: hermann.behre@medizin.uni-halle.de


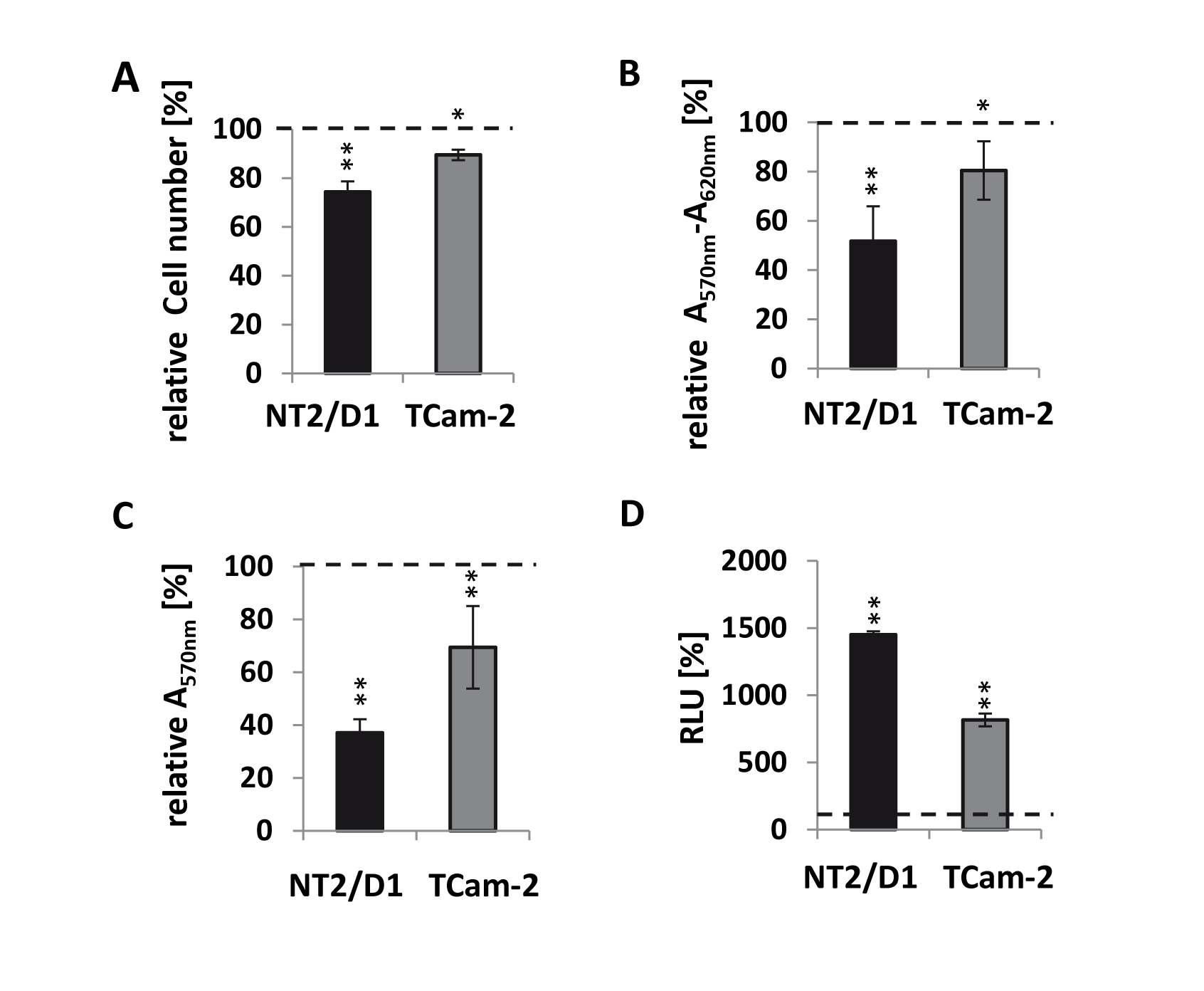


**Supplementary Figure 1.** Effects of a *PIWI-LIKE 2* knockdown on cell number **(A),** vitality **(B),** proliferation **(C)** and apoptosis induction **(D)** of NT2/D1 and TCam-2 cells in comparison to an untreated control of the respective cell line (dashed line). **(A)** **Number of cells** after *PIWI-LIKE 2* knockdown was measured after 48 h of cultivation of 200,000 cells in a Countess Cell counter (Invitrogen) subsequent to incubation with Trypan blue stain (Invitrogen). Both cell lines exhibited a slight, but significant decrease in cell number after *PIWI-LIKE 2* knockdown (NT2/D1: ‑25.7 %; TCam-2: -10.5 %, in comparison to untreated controls). **(B)** **Cell vitality:** Cells with *PIWI-LIKE 2* knockdown were cultured for 48 h and when fixed by trichloroacetic acid (Applichem). Fixed cells were stained with 4.4% sulforhodamine B (SRB, Sigma-Aldrich) solution, washed in 1% acetic acid and dried. For the measurement, the SRB stain was eluted in 20 nM Tris, pH 10 and extinction measured on a photospectrometer at 570 nm. Both, in NT2/D1 and TCam-2, the knockdown of *PIWI-LIKE 2* resulted in a significant reduction of cell vitality (NT2/D1: -62.9 %, TCam-2: -30.6 %, in comparison to untreated controls). **(C)** **Proliferation of cells** with *PIWI-LIKE 2* knockdown was measured by MTT assay (Promega). After 48 h of cultivation, cells were treated with 15 µl MTT dye solution, incubated for 4 h at 37°C and then treated with a solubilization/stop solution. The concentration of the formazan product was quantified at 570 nm at a photometer. Both cell lines exhibited a significant decrease in cell proliferation upon *PIWI-LIKE 2* knockdown (NT2/D1: -48.2 %, TCam-2: -19.6 %; in comparison to untreated controls). **(D) Apoptosis** induction was quantified by caspase 3/7-glo assay (Promega). Cells with *PIWI-LIKE 2* knockdown were cultured for 48 h, than treated with caspase 3/7 substrate solution. Luminescence was measured on a photometer 20 minutes after substrate addition. Both *PIWI-LIKE 2* knockdown cell lines showed a significant apoptosis induction (NT2/D1: +1351 %, TCam-2: +716 %, in comparison to untreated controls).

Student’s t-test, n = 3; *p <0.05, **p<0.01; Abbreviation: RLU = relative luminescence units.


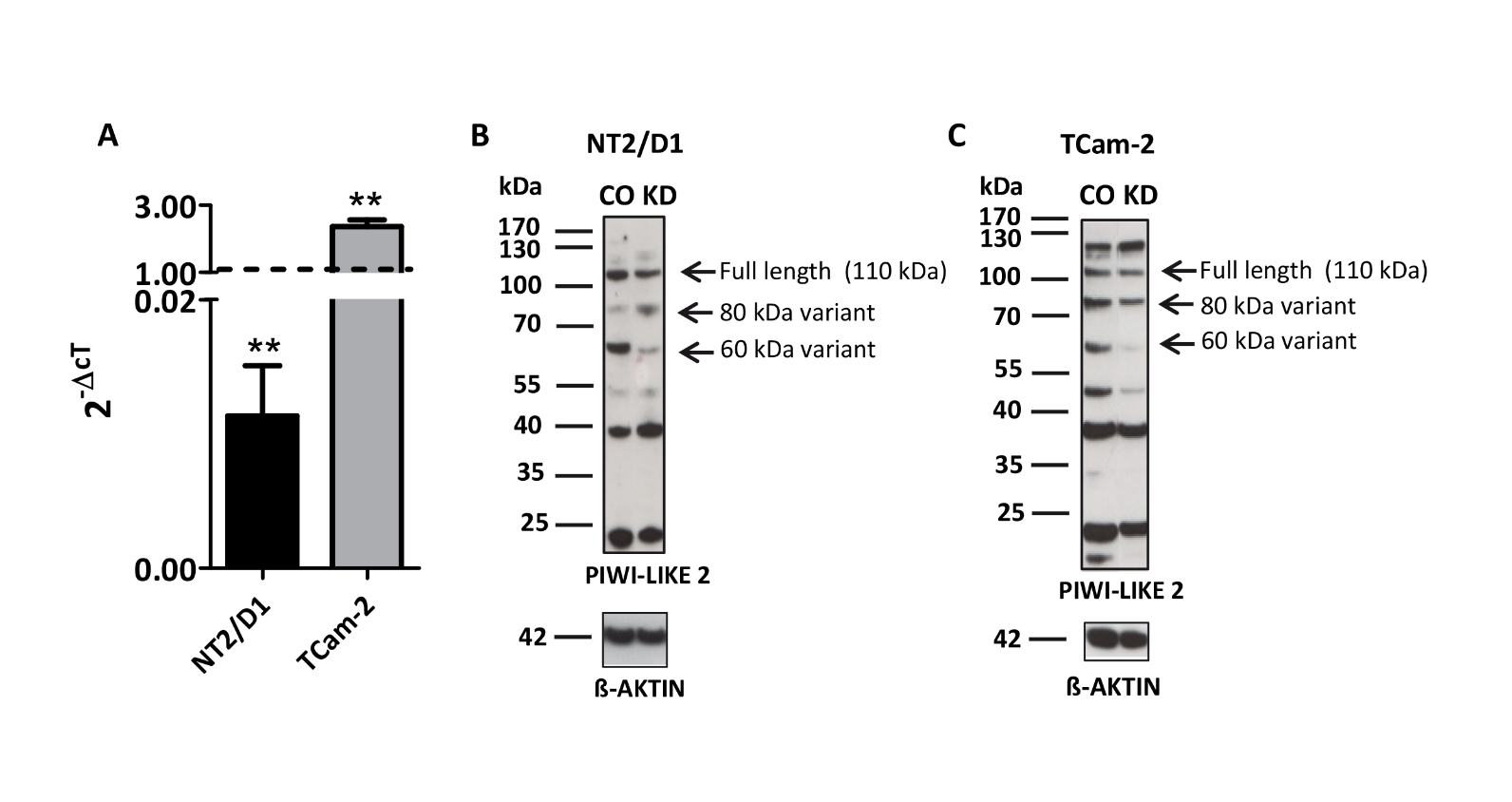


**Supplementary Figure 2.** Knockdown efficiency of *PIWI-LIKE 2* in NT2/D1 and TCam-2 cells on mRNA **(A)** and protein **(B,C)** level. **(A)** mRNA expression of *PIWI-LIKE 2* was measured by quantitative real-time PCR (qPCR). While *PIWI-LIKE 2* transcripts were significantly downregulated in NT2/D1, a 2fold induction of *PIWI-LIKE 2* transcripts was detected in TCam-2. **(B,C)** However, in Western Blots, the downregulation of the PIWI-LIKE 2 protein full length variant as well as the downregulation of two relevant splice variants (80 kDa and 60 kDa) could be shown in both NT2/D1 and TCam-2. ** p < 0.01; Student’s t-test; Abbreviations: CO = control; KD = *PIWI-LIKE 2* knock-down
